# The Characteristics of Ubiquitous and Unique *Leptospira* Strains from the Collection of Russian Centre for Leptospirosis

**DOI:** 10.1155/2014/649034

**Published:** 2014-09-02

**Authors:** Olga L. Voronina, Marina S. Kunda, Ekaterina I. Aksenova, Natalia N. Ryzhova, Andrey N. Semenov, Evgeny M. Petrov, Lubov V. Didenko, Vladimir G. Lunin, Yuliya V. Ananyina, Alexandr L. Gintsburg

**Affiliations:** N.F. Gamaleya Institute for Epidemiology and Microbiology, Ministry of Health of Russia, Gamaleya Street 18, Moscow 123098, Russia

## Abstract

*Background and Aim. Leptospira*, the causal agent of leptospirosis, has been isolated from the environment, patients, and wide spectrum of animals in Russia. However, the genetic diversity of* Leptospira* in natural and anthropurgic foci was not clearly defined.* Methods*. The recent MLST scheme was used for the analysis of seven pathogenic species. 454 pyrosequencing technology was the base of the whole genome sequencing (WGS).* Results*. The most wide spread and prevalent* Leptospira* species in Russia were* L. interrogans, L. kirschneri,* and* L. borgpetersenii*. Five STs, common for Russian strains: 37, 17, 199, 110, and 146, were identified as having a longtime and ubiquitous distribution in various geographic areas. Unexpected properties were revealed for the environmental* Leptospira *strain Bairam-Ali. WGS of this strain genome suggested that it combined the features of the pathogenic and nonpathogenic strains and may be a reservoir of the natural resistance genes. Results of the comparative analysis of* rrs* and* rpoB* genes and MLST loci for different* Leptospira* species strains and phenotypic and serological properties of the strain Bairam-Ali suggested that it represented separate* Leptospira* species.* Conclusions*. Thus, the natural and anthropurgic foci supported ubiquitous* Leptospira* species and the pool of genes important for bacterial adaptivity to various conditions.

## 1. Introduction

*Leptospira* is a genus of bacteria that is encountered in all geographical areas, except arctic and arid regions. Some* Leptospira* are responsible for leptospirosis, a natural-focus disease. According to the List of Prokaryotic names with Standing in Nomenclature, the* Leptospira* genus has 21 species [1]. Seven of the species have been established as pathogenic:* L. interrogans*,* L. borgpetersenii*,* L. kirschneri*,* L. noguchii*,* L. santarosai*,* L. weilii*, and* L. alexanderi*. Two species,* L. alstonii* and* L. kmetyi*, are candidates for assignment to the pathogenic group; they are rarely isolated, and the sources of their isolation are not ill humans.* L. alstonii* was isolated from a frog and* L. kmetyi* was isolated from soil. Phylogenetic analysis with 16S rRNA gene sequences showed that* L. alstonii* and* L. kmetyi* clustered with the pathogenic* Leptospira* species [2, 3]. Five species,* L. broomii, L. fainei, L. inadai, L. licerasiae,* and* L. wolffii,* are classified as intermediate, and six species,* L. biflexa, L. meyeri, L. terpstrae, L. vanthielii, L. wolbachii, *and* L. yanagawae, *are nonpathogenic [4]. One new species,* L. idonii*, isolated from the environmental water, is candidate for assignment to the nonpathogenic group. This species is placed within the clade of the known saprophytic species of the genus* Leptospira* on the 16S rRNA gene-based phylogenetic analysis [5].

According to WHO guidance [6], the incidence of leptospirosis ranges from about 0.1–1 per 100 000 persons per year in temperate climates to 10–100 per 100 000 in the humid tropics. In the Russian Federation, only 0.01 cases per 100 000 were reported in 2013. During 2012-2013, 506 cases were registered in Russia according to the report of the Federal Service for Supervision of Consumer Rights Protection and Human Welfare (http://rospotrebnadzor.ru/). Long-term control of the multiple natural and anthropurgic foci in the USSR has been organized with the participation of the MoH Centre for Leptospirosis, an action which may be responsible for the decrease in incidence. During the registration period, strains of* Leptospira* species were isolated, from the animals (maintenance and supplementary hosts of the* Leptospira*), human patients, and environment, and saved in the Gamaleya Institute Microbial Collection (GIMC).

Among the strains of* Leptospira* species in GIMC one strain was mysterious and not closely related to any* Leptospira*, either pathogenic or saprophytic; it was named* Leptospira *spp. strain Bairam-Ali. Bairam-Ali was isolated from the water of a drainage canal in Turkmenia in 1971. On the basis of phenotypic tests, it was classified as a saprophytic species, although its resistance to the leptospiracidal activity of normal mammal sera and some other features made it closer to the pathogenic leptospires. Thus,* Leptospira *sp. strain Bairam-Ali was used in a diagnostic system on the basis of macroagglutination reaction. This diagnostic system was different from the original genus-specific microagglutination test, which requires multiple serovars of live* Leptospira* and involves a risk of infection. Strain Bairam-Ali is a natural substitute for the microcapsule of synthetic polymer [7] as carrier of antigens similar to pathogenic strains, and it is safe for humans.

Only whole genome sequence could help resolve the mystery of the strain Bairam-Ali and clarify its phylogenetic position in the* Leptospira* genus and relationships with the pathogenic* Leptospira* species.

## 2. Materials and Methods 

### 2.1. Bacterial Strains

*Leptospira* strains were cultured by the Russian MoH Centre for Leptospirosis laboratory at the N.F. Gamaleya Institute for Epidemiology and Microbiology, Moscow, according to WHO guidance [6]. Fifty-eight strains, including 29 reference strains and 29 isolates from various sources and geographical regions, were analyzed. Twenty-six reference strains were members of seven pathogenic species. By the start of this study, seven reference strains were absent from the* Leptospira* MLST database [8].

### 2.2. Phenotypic and Serological Characterization of Strain Bairam-Ali

Methods for differentiation of pathogenic versus saprophytic strains and for cross-agglutination-absorption reactions were performed according to WHO guidance [6].

### 2.3. Scanning Electron Microscopy

Samples were prepared as described in detail [9] and analyzed with dual-beam focused ion beam/scanning electron microscope, Quanta 200 3D (FEI Company, USA), in both high and low vacuum, mostly at 5 kV electron beam acceleration [10].

### 2.4. DNA Isolation

DNA for PCR analysis was extracted from the bacterial cultures as described previously [11]. Preparation of genomic DNA for the whole genome sequencing was performed according to [12].

### 2.5. *Leptospira* Species Identification

The species of isolates were identified by amplification and sequencing of the* rpoB* gene (coding *β* subunit of bacterial RNA polymerase), according to La Scola et al. [13]. Sequence data for* rpoB* has been deposited in GenBank, with accession numbers KJ701730–KJ701749.

### 2.6. MLST

MLST for the strains of* L. interrogans* and* L. kirschneri* was performed by use of the original scheme of Thaipadungpanit et al. [14]. After publication of the modified MLST scheme [15], we completed earlier results by using* caiB* gene sequences. For the new isolates and strains of five other species, we used only the modified MLST scheme. Some modifications were inserted in the published protocol. The conditions of the amplification were modified for the* glmU*,* pntA*, and* sucA* genes by raising the melting temperature to 50°C. Also, the MgCl_2_ concentration was changed to 3.5 mM for the* glmU*,* pntA*, and* sucA* genes. Reference collection strains were used for adaptation of the method to our laboratory and for control of the reproducibility of the results.

### 2.7. PCR Products Sequencing

PCR products were sequenced according to the protocol of BigDye Terminator 3.1 Cycle Sequencing kit for the Genetic Analyzer 3130 of Applied Biosystems/Hitachi.

### 2.8. Nucleotide Sequence Analysis

The alignment of* rpoB* and MLST gene sequences was made by use of ClustalW2 [16]. BLAST search was used for species identification; similarity of* rpoB* gene sequences was more than 98%.

Allele numbers for MLST genes were assigned with the help of website MLST Home. Allelic profiles, in the order glmU-pntA-sucA-tpiA-pfkB-mreA-caiB, were used to assign sequence types (STs) to strains. New alleles and ST were controlled and submitted by the curator of* Leptospira *spp. MLST database [8]. All new* Leptospira* strains were submitted in the* Leptospira *spp. MLST database under the identification numbers indicated in Table 1.

Relatedness between STs on the base of allelic profiles was analyzed by use of BURST version 1.00 [17, 18].

### 2.9. Nucleotide Sequence Polymorphism

BLAST search was used for retrieving homologues* rrs *(16S rRNA-coding)*, rpoB, *and MLST gene sequences from GenBank (http://www.ncbi.nlm.nih.gov/genome/browse/). For comparative sequence analysis and phylogenetic reconstruction, nucleotide sequences of 92 additional* Leptospira* strains were retrieved. Seventy-five nucleotide sequences represented either complete or partial cds of* rrs* gene and* rpoB* gene, and 15 sequences represented whole genome sequencing data and genome drafts (Table 1).* Turneriella parva* have been included in the analysis as an out-group taxon from* Leptospiraceae*, for which whole genome sequence data are available (Table 1). Sequences of seven concatenated MLST loci for 201 ST available at the time of analysis were retrieved [8]. The alignments of* rrs, rpoB,* and MLST gene sequences and analysis nucleotide similarity (in %) were performed by use of ClustalW2 [16].

### 2.10. Phylogenetic Analysis

Phylogenetic analysis of* Leptospira* species was performed based on the* rrs* gene fragment,* rpoB* gene fragment, and seven concatenated sequences of MLST loci. Phylogenetic trees were constructed by use of neighbor-joining, maximum likelihood, and maximum parsimony methods.

Genetic distances between* Leptospira *genotypes were evaluated by use of Kimura's two-parameter model [19], which was chosen as an optimal evolution distance model derived from model test based on the Akaike information criterion [20]. The evolutionary history was inferred by using the Maximum Likelihood method based on the general time reversible model [21]. Initial tree(s) for the heuristic search were obtained automatically by applying neighbor-joining and BioNJ algorithms to a matrix of pairwise distances estimated by use of the maximum composite likelihood approach and then selecting the topology with superior log likelihood value. A discrete gamma distribution was used to model evolutionary rate differences among sites (six categories (+G, parameter = 0.4818)). Maximum parsimony trees were constructed with an algorithm implemented in MEGA version 6.0 [22]. Bootstrap analyses were performed with 1,000 replicates.

### 2.11. Whole Genome Sequencing

Whole genome sequencing of* Leptospira *spp. strain GIMC2001:Bairam-Ali was performed according to the manufacturer's guidelines (Roche) for the next generation sequencing (NGS). Two protocols were used for a shotgun-sequencing library preparation: rapid library and pair-end library. The rapid library was made according to the Rapid Library Preparation Method Manual (Roche). The pair-end library was performed according to the 3 kb protocol provided by the manufacturer to aid in scaffold building. The paired-end library insert size was from 1347 to 4364 bp, with an average of 2695 bp.

Assembly was performed with 454 Sequencing System Software v.2.7 (Roche), yielding 14 scaffolds, with the largest size being 3 342 467 bp. Gap closure was performed by use of Contig Graph result file generated by GS De Novo Assembler program (Roche). For the oriC region search, Ori-Finder program was used [23].

### 2.12. Gene Annotation

The software Rapid Annotations Subsystems Technology and SEED [24, 25] were used for annotating the genome of* Leptospira *spp. strain GIMC2001:Bairam-Ali.

## 3. Results and Discussion

A sample of the collection (GIMC) of the Russian MoH Centre for Leptospirosis was used for the verification the species and strains based on the currently recommended molecular-genetic methods. The sample included 29 reference strains and 29 isolates of* Leptospira *from various geographical regions (Table 1,  *—marker of the reference strains from GIMC).

The analysis of* rpoB* gene sequences demonstrated that 29 reference strains belonged to seven pathogenic species, one nonpathogenic species (*L. yanagawae*), and one intermediate species (*L. inadai*). Among 29 isolates of* Leptospira *twenty-four field isolates were related to the three pathogenic species (*L. interrogans*,* L. borgpetersenii,* and* L. kirschneri*). Four cultures were isolated from the commercially available Ellinghausen-McCullough-Johnson-Harris (EMJH) medium: two isolates from this group belonged to the nonpathogenic species* L. biflexa* and the other two isolates belonged to the intermediate species* L. inadai*. Thus, the prevailing species among the isolates collected on the territory of Russia comprised* L. interrogans*,* L. borgpetersenii,* and* L. kirschneri*.

The modified MLST scheme was applied to the isolates and reference strains representing 7 pathogenic* Leptospira* species. Twenty-four isolates of pathogenic species belonged to 8 different STs (Table 1). Five STs, common for Russian strains of* Leptospira* (*L. interrogans* ST37, 17, and 199;* L. kirschneri* ST110; and* L. borgpetersenii* ST146), were identified as having a longtime and ubiquitous distribution in various geographic areas.

Among the strains of* Leptospira* species available in GIMC, one strain seemed to be mysterious and not closely related to any* Leptospira*, either pathogenic or saprophytic, or intermediate. It was named* Leptospira *spp. strain Bairam-Ali, because it was isolated in 1971 in Turkmenia from the water of a drainage canal.

### 3.1. Morphology of the* Leptospira* spp. Strain Bairam-Ali

**Figure 1:**
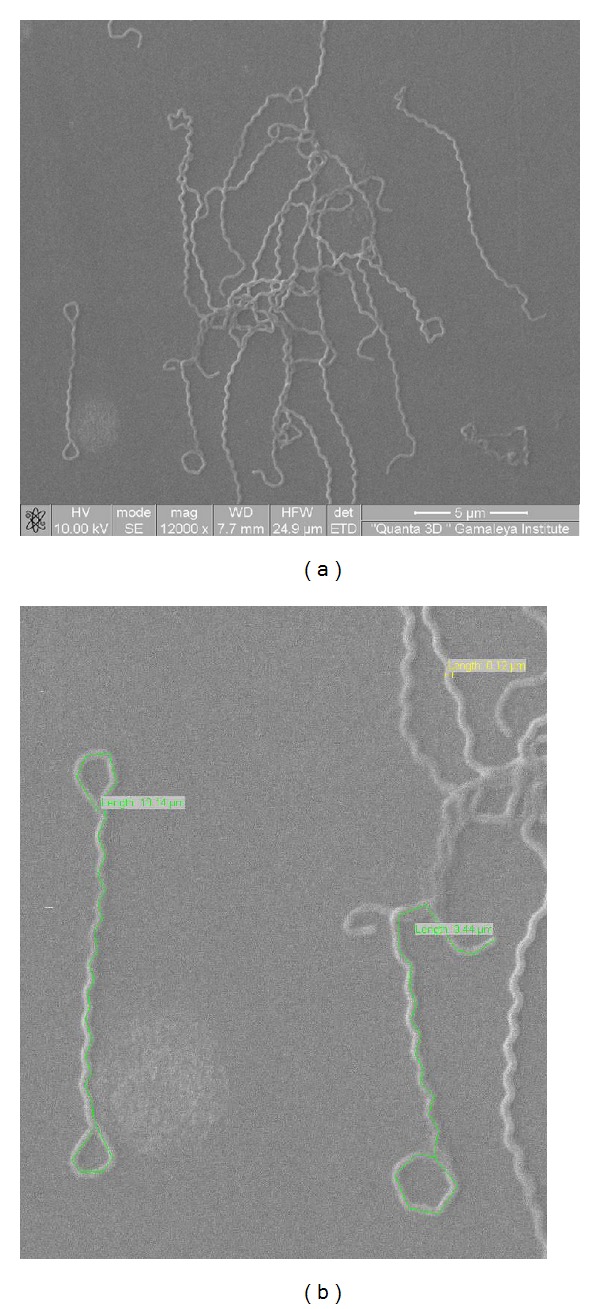
Dual-beam scanning electron microscopy image of* L. *species Bairam-Ali cells. (a) Original image; (b) with measuring the object in the program “Scandium” (green, length 10,14 *μ*m; yellow, length (diameter) 0,12 *μ*m; and green, length 9,44 *μ*m).

The morphology of the mysterious strain Bairam-Ali is typical of that of the* Leptospira* genus (Figure 1). Electron microscopy demonstrated that its cells are corkscrew-shaped with end hooks. They are thin and helical, like the cells of all known leptospires. Also, the cells have a diameter (*d*) of 0.12 *μ*m and length (*l*) from 9.44 to 10.14 *μ*m, like that of known leptospires (*d* = 0.15–0.3 *μ*m and *l* = 6,00–20,00 *μ*m) [26].

### 3.2. Phenotypic Characterization of the* Leptospira* spp. Strain Bairam-Ali

Physiological characteristics of Bairam-Ali, demonstrated in Table 2, suggest that strain Bairam-Ali can be classified as saprophytic* Leptospira*.

For pathogenicity experiment, six four-week-old male golden Syrian hamsters were inoculated subcutaneously with 10^8^ cells of strain Bairam-Ali in 1 mL of PBS. No hamster died from infection even at such a high bacterial dose.* Leptospira* cells were not detected during following bacterioscopic and bacteriological examination of the hamsters' viscera. This is an additional evidence of saprophytic quality of Bairam-Ali.

On the other hand, Bairam-Ali was resistant to the bactericidal (leptospiracidal) activity of the normal serum of human and of some other mammal animals. The most of pathogenic* Leptospira* species were resistant too, whereas the saprophytic species* L. biflexa* was sensitive.

### 3.3. Serological Characterization of the* Leptospira* spp. Strain Bairam-Ali

The strain Bairam-Ali had no antigenic affinity with 18 different serovars represented by reference strains. So the conclusion about the original serotype of Bairam-Ali was made. The serovar of Bairam-Ali was named bairam-ali. It formed the separated serogroup Bairam-Ali.

### 3.4. The Whole Genome Sequence Analysis

The whole genome sequence could help to resolve the mystery of the strain Bairam-Ali and clarify its phylogenetic position in the* Leptospira* genus.

According to the NGS data the whole Bairam-Ali genome is 4,4 Mb, with GC % 34,74 and two chromosomes. The first chromosome is 4 059 463 bp and the second is 325 649 bp. Plasmid, typical of nonpathogenic* Leptospira* species, was absent from the Bairam-Ali genome. On the base of genome size and GC content, demonstrated in Table 3, Bairam-Ali is more similar to the pathogenic species* L. interrogans* and* L. noguchii*.

Based on* rpoB *gene sequence, we established that Bairam-Ali is more similar to pathogenic strain* L. interrogans *Fiocruz-L1-130 (but the level of similarity is only 70.00%). However, by sequence of* rDNA *genes it is similar to nonpathogenic strains: for gene* rrs*, coding 16S rRNA,* L. meyeri* was most closely related (92.74%); for gene* rrl,* coding 23S rDNA,* L. biflexa* was more similar (95.00% fragment 1 and 93.00% fragment 2). Sequences of* rDNA* genes of Bairam-Ali were deposited in GenBank, accession numbers KJ701750, KJ701751, and KJ701752.

These data suggest dual phenotypic characteristics of the strain Bairam-Ali. In spite of having differences from both pathogenic and nonpathogenic species, many of the functional categories that are involved in essential housekeeping functions are represented in its core gene [27, 28]. Thus, basic groups of proteins involved in cell metabolism, survival, environmental adaptability, and potential pathogenic factors were present. Enzyme complexes participating in implementation of genetic information, in particular in DNA replication, were identified in strain Bairam-Ali: chromosomal replication initiator protein DNA, single-stranded DNA-binding protein; all subunits of DNA polymerase III, DNA polymerase I, and DNA polymerase IV; DNA gyrase; ligase; and helicase [29]. Also, the large groups of enzymes that take part in DNA reparation [29], for example, excinuclease ABC subunits A–C and exodeoxyribonuclease (III, V, and VII), and proteins of postreplicative mismatch repair system (Mutator S and Mutator L) were present. Archaeal DNA polymerase I gene was detected in the Bairam-Ali genome; this gene is a member of Family B and bacterial DNA polymerase II. The same gene was present in nonpathogenic strain* L. biflexa* Patoc genome but not in pathogenic* L. interrogans *Fiocruz-L1-130 and* L. borgpetersenii *Hardjo-bovis-L550 genomes [27, 30]. The sequences of DNA metabolome have been deposited in GenBank, with accession numbers KJ701710–KJ701729.

According to Bourret et al. [31], enteric bacteria usually have about 50 genes coding for structural and functional proteins involved in motility. Motility is a distinctive feature of* Leptospira*; forty-seven different proteins provide bacterial locomotion [32]. In the Bairam-Ali genome, the genes for proteins of the basic parts of periplasmic flagella were detected: basal-body (Flg B–D, FlgF, and FlgG), hook (FlgE, FlgK, FlgL, FliE, FliD, and fliK), and four copies of FlaA (sheath protein of filament). The genes for proteins of flagellar rings L (FlgH), MS (FliF), and P (FlgI); biosynthesis protein (FlhA, FlhB (2), FlhF, FliL, and Fli Q-S); motors (MotB (3), MotA (2), FliG (3), FliM, and FliN); flagellar assembly factor (FliW and FliH), cell division (BolA (2), FtsA, FtsH (2), FtsI, FtsK, FtsW, and FtsZ), and gliding motility (GldF and GldG) proteins also were registered. Strain Bairam-Ali was found to have more than ten genes for the regulation of flagellum genes transcription and for signal transduction to flagella motor. Sequences of the flagellum genes have been deposited in GenBank, accession numbers KJ701653–KJ701709.

It should be noted that the important structural element, that is, numerous groups of predicted lipoproteins (Lip), which may be either surface-exposed or located in the periplasm, is present in both saprophytic and pathogenic* Leptospira *species. In the strain Bairam-Ali genome, as in the genome of nonpathogenic species* L. biflexa* [27], genes or orthologs of the major lipoproteins LipL32 and LipL41, which are important immunodominant antigens in pathogenic* Leptospira *species [33, 34], were not identified. Nevertheless, in the genome of Bairam-Ali, we detected six genes of LipL45 (GenBank accession numbers KJ701647–KJ701652), which are processed to a peripheral membrane associated with the outer membrane complex. According to Matsunaga et al. [35] expression of P31, derived from the carboxy terminus of LipL45, is upregulated in stationary phase cultures; thus it may have a membrane-stabilizing function. Also, in a hamster model infected with pathogenic* L. kirschneri*, antibodies to LipL45 [35] were produced, suggesting its involvement in pathogenesis.

Further analysis of the genome of Bairam-Ali revealed the presence of genes for more than 40 different classes of proteins, ensuring its natural resistance to a wide range of antibiotics (*β*-lactams, tetracyclines, glycopeptides (vancomycin), and polymyxin); efflux system and resistance to heavy metals (Czc A–C and arsenical-resistance proteins); and possible abortive infection phage-resistance protein. The broad resistance characteristics of strain Bairam-Ali are consistent with it being a natural reservoir for storage and possible transmission of these properties to other free-living* Leptospira *species. Sequences of the resistome genes and their proteins have been deposited in GenBank, accession numbers KJ701605–KJ701646.

### 3.5. Sequence Polymorphism

To determine the place of the original strain Bairam-Ali in the* Leptospira* genus, we undertook phylogenetic analysis of the* Leptospira* groups with different pathogenicity.

Based on performed alignments, the percent nucleotide similarities of* rrs, rpoB,* and MLST gene sequences of analyzed* Leptospira* genotypes were determined; see Supplementary Material available online at http://dx.doi.org/10.1155/2014/649034 (S1, S2, and S3). According to the obtained data, aligned sequences of 16S rRNA-coding* rrs* gene were 1305 bp, aligned sequences of gene* rpoB* were 493 bp, and aligned sequences of seven MLST tags (*glmU-pntA-sucA-tpiA-pfkB-mreA-caiB*) were 3105 bp.

Among the investigated* Leptospira* sequences, gene* rpoB* showed the highest variability, reaching 40.61%. 16S rRNA-coding gene was more conservative, and general sequence variability of the* rrs *gene across all strains and species reached 11.52%. Sequence variability of seven MLST tags reached 34.88%.

Detectable intraspecific sequence polymorphism for studied loci also was different. Intraspecific differences detected by* rrs *were less than 1%. Intraspecific differences detected by most polymorphic* rpoB* genes ranged from 0.61% (*L. kirschneri*) to 11.63% (*L. borgpetersenii*), with, in some cases, overlapping of intraspecific and interspecific values (S1, S2, and S3).

Notably, the intraspecific differences detected by seven MLST tags resolved all of the* Leptospira* genotypes and matched 3% accepted threshold values for prokaryote species divergence [36, 37]. The exception was* L. weilii*; interspecies differences of* L. weilii* strains revealed by seven MLST tags reached 5.77% (S1, S2, and S3).

Despite the differences in resolution ability of studied loci, all of them confirmed the unique nature of* Leptospira* spp. strain Bairam-Ali.* rrs*,* rpoB*, and MLST tags obtained for Bairam-Ali had low similarity with other sequences. Maximum similarity of Bairam-Ali MLST tags (69.7%) was detected for* L. interrogans* (Table 3). At the same time, related levels of sequence similarity also characterized nonpathogenicand pathogenic* Leptospira* species (67.29–68.95%) and intermediate and pathogenic* Leptospira* species (70.0–72.8%). Sequence similarity in pairs of Bairam-Ali, to intermediate* Leptospira* species, to nonpathogenic* Leptospira *species, and to pathogenic* Leptospira* species, was 65.8–67.31%, 67.6–69.18%, and 66.27–69.7%, respectively. The level of sequence similarity between Bairam-Ali and* Turneriella parva *was the lowest (55.3%), which is evidence of Bairam-Ali belonging to the genus* Leptospira.*

### 3.6. *Leptospira* Phylogeny and* L.* spp. Bairam-Ali Location in the* Leptospira* Genus

The most prominent phylogenetic information was obtained through concatenated sequences of seven MLST tags. All genotypes were divided into three species groups: pathogenic, nonpathogenic, and intermediate.* T. parva* formed a distinct basal out-group branch.

**Figure 2:**
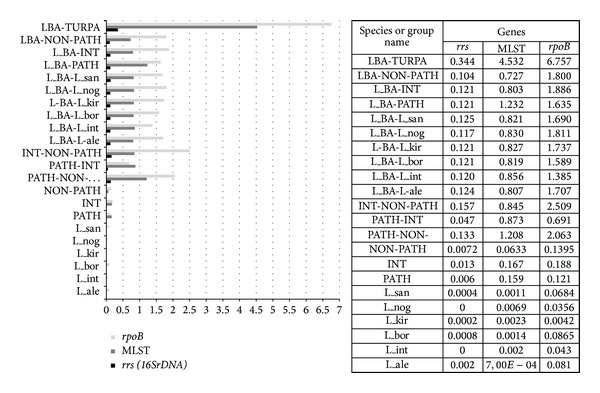
The number of base substitutions per site from averaging over all sequence pairs within each group is shown. Analyses were conducted using the Kimura 2-parameter model.

The genetic diversity of pathogenic* Leptospira* reached 0.159 (MLST data), 0.006 (*rrs* data), and 0.121 (*rpoB* data). The most variable species were* L. borgpetersenii *(0.086* rpoB*) and* L. noguchii* (0.069). Despite the fact that* L. interrogans* was the most representative species in the analysis (120 MLST genotypes), intraspecies genetic diversity for* L. interrogans* was only 0.002 (Figure 2).

The genetic diversity of nonpathogenic* Leptospira *was similar to that of the pathogenic* Leptospira *species and reached 0.063 (MLST data), 0.007 (*rrs* data), and 0.139 (*rpoB* data).

Interestingly, the intermediate group* Leptospira* had broader genetic diversity than did the nonpathogenic or pathogenic* Leptospira* (0.167, MLST data; 0.013,* rrs *data; and 0.188* rpoB *data) (Figure 2).

In pathogenic group accessions of each species, excluding* L. weilii,* clearly distinct phylogenetic clusters, with high bootstrap supporting values, were present.In some data sets, significant subdivision of* L. weilii* genotypes, combined with a close relationship between* L. weilii* and* L. alexanderi, *was found. Possible polyphyletic nature of* L. weilii* has been described previously, particularly in the highly divergent* tpiA* locus [15]. According to our data, excluding from analysis* glmU* and* pfkB* loci,* L. weilii* and* L. alexanderi *genotypes fall into two different but closely related subclusters. This differentiation was found also with* rrs* gene phylogeny (S4).

**Figure 3:**
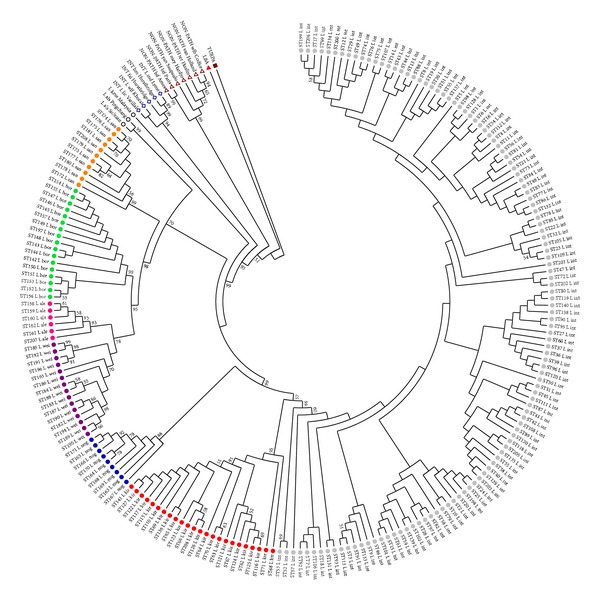
Phylogenetic tree of* Leptospira* species based on concatenated sequences of seven MLST loci.

Three species in the pathogenic group,* L. interrogans, L. kirschneri, *and* L. noguchii,* formed one close genetic subgroup (BI = 99%, bootstrap index). The strains of these species are often the cause of the human leptospirosis. Four species,* L. weilii, L. alexanderi, L. borgpetersenii, and L. santarosai, *formed another phylogenetic subgroup (BI = 98%) (Figure 3). The strains of these species are usually isolated in natural foci. The candidate pathogenic species,* L. alstoni* and* L. kmetyi,* fell in the common big cluster of pathogenic* Leptospira* species. On the MLST tree,* L. alstoni *formed a sister clade to the* L. weilii/L. alexanderi/L. borgpetersenii/L. santarosai *group of pathogenic* Leptospira* species (BI = 70%), whereas* L. kmetyi *formed a basal branch (BI = 99) (Figure 3). The levels of genetic similarity of* L. alstoni* and* L. kmetyi *to pathogenic* Leptospira* species (0.913 and 0.926) were much higher than to nonpathogenic (0.13 and 0.09) or intermediate species (0.58 and 0.54). Our data confirm the results of previous characterization for* L. alstoni* on the base of* 16SrDNA* gene and for* L. kmetyi* on the base of* 16SrDNA, gyrB*, and* rpoB* genes analysis [2, 3].

The single* Leptospira* spp. strain, Bairam-Ali, formed a separate distinct branch on dendrogram, with genetic distances comparable to those of distantly related species of different* Leptospira *groups. For example, differences between pathogenic and nonpathogenic, pathogenic and intermediate, and intermediate and nonpathogenic groups were 1.208, 0.873, and 0.845, respectively (Figure 2), whereas the differences between* Leptospira* spp. Bairam-Ali and pathogenic, intermediate, and nonpathogenic species were 1.232, 0.803, and 0.727, respectively; this result points to significant differences between Bairam-Ali and other* Leptospira* species, a result that is consistent with whole genome sequencing data.

It should be noted that the comparative studies of different* Leptospira *groups based on the* rrs* gene variability, which added to the analysis the newly described saprophytic* L. idonii* strain Eri-1T [5] and recently approved 16S rDNA sequences of leptospires from the Peruvian Amazon, previously termed “clade C” [38, 39], did not change separate phylogenetic position of* Leptospira* spp.strain Bairam-Ali.

Thus, according to our results the* Leptospira* strain Bairam-Ali forms a separate phylogenetic branch of the* Leptospira* genus and cannot be attributed to any group of pathogenic, intermediate, or saprophytic* Leptospira*, illustrating its uniqueness.

In conclusion, we found that the natural and anthropurgic foci supported ubiquitous pathogenic, intermediate, and nonpathogenic* Leptospira* species. They have circulated for a long time, interacting with maintenance and supplementary animal hosts. The relationships between the different* Leptospira* strains provide for horizontal gene transfer and create in the bacterial population the pool of genes important for adaptivity to various conditions. Modern molecular genetic methods are suitable for investigation of* Leptospira* and control of the pathogenic species, which are the causal agent of leptospirosis. Also, the taxonomic position of the new strains with unexpected properties can be founded on the base of these methods. Further investigation of the genetic diversity in the natural and anthropurgic foci is required for the identification of the* Leptospira *genotypes, control of the strain's transmission, and better understanding of the origin and evolution of the* Leptospira* species.

## Supplementary Material

Supplementary Table S1: The range of nucleotide sequences similarity (rrs data) between and within groups of Leptospiraceae samples.
Supplementary Table S2: The range of nucleotide sequences similarity (rpoB data) between and within groups of Leptospiraceae samples.
Supplementary Table S3: The range of nucleotide sequences similarity (MLST data) between and within groups of Leptospiraceae samples.
Supplementary Figure S4: Phylogenetic tree of Leptospira species based on 16S rDNA sequences.

## Figures and Tables

**Table 1:** Strains used in genotyping and phylogenetic analysis.

Number	Strain	Species	ST	Our submission ID or GenBank accession number for MLST genes	rpoB GenBank accession number	16S rDNA GenBank accession number
1	∗GIMC2029:Li130	*L. alexanderi*, serogroup Manhao, serovar lichuan	207	337	KJ701742	
2	∗GIMC2051:Veldrat Bataviae 46	*L. borgpetersenii*, serogroup Javanica, serovar javanica	143	In MLST Home data base	KJ701733	
3	∗GIMC2052:Sari	*L. borgpetersenii*, serogroup Mini, serovar mini	142	In MLST Home data base	KJ701736	
4	GIMC2002:101PJ	*L. borgpetersenii*, serogroup Javanica, serovar poi	146	339		
5	GIMC2003:29PJ	*L. borgpetersenii*, serogroup Javanica, serovar poi	146	340	KJ701734	
6	GIMC2004:Yaroslavl 7	*L. borgpetersenii*, serogroup Javanica, serovar poi	146	341		
7	GIMC2005:1217PJ	*L. borgpetersenii*, serogroup Javanica, serovar javanica	146	342		
8	GIMC2006:1622PJ	*L. borgpetersenii*, serogroup Javanica	146	343		
9	GIMC2007:5-I	*L. borgpetersenii*, serogroup Javanica	146	344		
10	∗GIMC2049:Castellon 3	*L. borgpetersenii*, *serogroup Ballum, serovar castellonis *	149	In MLST Home data base	KJ701737	
11	∗GIMC2050:Perepelitsin	*L. borgpetersenii*, serogroup Tarassovi, serovar tarassovi	153	In MLST Home data base	KJ701735	
12	∗GIMC2008:Mus 24	*L. borgpetersenii*, serogroup Sejroe, serovar saxkoebing	155	345		
13	∗GIMC2048:Naam	*L. interrogans*, serogroup Icterohaemorrhagiae, serovar naam	23	In MLST Home data base	KJ701731	
14	GIMC2014:AV4	*L. interrogans*, serovar no data	23	351		
15	∗GIMC2047:Djasiman	*L. interrogans*, serogroup Djasiman, serovar djasiman	11	In MLST Home data base		
16	∗GIMC2046:RGA	*L. interrogans*, serogroup Icterohaemorrhagiae, serovar icterohaemorrhagiae	17	In MLST Home data base		
17	GIMC2010:Sv-19	*L. interrogans*, serogroup Icterohaemorrhagiae	17	347		
18	GIMC2009:Rn-2010	*L. interrogans*, serogroup Icterohaemorrhagiae, serovar copenhageni	17	346		
19	GIMC2011:Rn-493	*L. interrogans*, serogroup Icterohaemorrhagiae, serovar copenhageni	17	348		
20	GIMC2012:Rn-16	*L. interrogans*, serogroup Icterohaemorrhagiae, serovar copenhageni	17	349		
21	GIMC2013:Rn-77	*L. interrogans*, serogroup Icterohaemorrhagiae, serovar copenhageni	17	350		
22	∗GIMC2042:Ezh-1 = Jez Bratislava	*L. interrogans*, serogroup Australis, serovar bratislava	24	In MLST Home data base		
23	∗GIMC2043:Akiyami A	*L. interrogans*, serogroup Autumnalis, serovar autumnalis	27	In MLST Home data base		
24	∗GIMC2044:Zanoni	*L. interrogans*, serogroup Pyrogenes, serovar zanoni	31	In MLST Home data base	KJ701732	
25	∗GIMC2045:Hebdomadis	*L. interrogans*, serogroup Hebdomadis, serovar hebdomadis	36	In MLST Home data base		
26	GIMC2015:Kashirsky	*L. interrogans*, serogroup Canicola, serovar canicola	37	352		
27	GIMC2016:Mitronov	*L. interrogans*, serogroup Canicola, serovar canicola	37	353		
28	GIMC2017:Bugay	*L. interrogans*, serogroup Canicola, serovar canicola	37	354		
29	GIMC2018:Sobaka 2000	*L. interrogans*, serogroup Canicola, serovar canicola	37	355		
30	GIMC2019:Udalov	*L. interrogans*, serogroup Canicola, serovar canicola	37	356		
31	∗GIMC2038:Ballico	*L. interrogans*, serogroup Australis, serovar australis	51	In MLST Home data base		
32	∗GIMC2039:3705	*L. interrogans*, serogroup Sejroe, serovar woffi	58	In MLST Home data base		
33	∗GIMC2040:Szwajizak	*L. interrogans*, serogroup Mini	73	In MLST Home data base		
34	∗GIMC2041:Pomona	*L. interrogans*, serogroup Pomona, serovar pomona	140	In MLST Home data base		
35	∗GIMC2020:M-20R	*L. interrogans*, serogroup Icterohaemorrhagiae, serovar copenhageni	199	357	KJ701730	
36	GIMC2021:Abduloev	*L. interrogans*, serogroup Icterohaemorrhagiae, serovar copenhageni	199	358		
37	GIMC2022:CL-11	*L. interrogans*, serogroup Icterohaemorrhagiae, serovar copenhageni	199	359		
38	GIMC2030:CL-17	*L. interrogans*, serogroup Icterohaemorrhagiae, serovar copenhageni	206	336		
39	∗GIMC2023:Vleermuis 3868	*L. kirschneri*, serogroup Cynopteri, serovar cynopteri	70	360		
40	∗GIMC2024:Moskva V	*L. kirschneri*, serogroup Grippotyphosa, serovar grippotyphosa	110	361		
41	GIMC2025:181PG	*L. kirschneri*, serogroup Grippotyphosa, serovar grippotyphosa	110	362	KJ701738	
42	GIMC2026:617PG	*L. kirschneri*, serogroup Grippotyphosa, serovar grippotyphosa	110	363		
43	GIMC2027:859PG	*L. kirschneri*, serogroup Grippotyphosa, serovar grippotyphosa	110	364		
44	GIMC2028:1106PG	*L. kirschneri*, serogroup Grippotyphosa, serovar grippotyphosa	110	365		
45	∗GIMC2037:5621	*L. kirschneri*, serogroup Pomona, serovar mozdok	117	In MLST Home data base		
46	∗GIMC2031:HS 26R	*L. kirschneri*, serogroup Bataviae, serovar djatsi	204	334	KJ701739	
47	∗GIMC2033:LSU 1945	*L. noguchii*, serogroup Louisiana, serovar panama	169	In MLST Home data base	KJ701740	
48	∗GIMC2034:CZ 214 K	*L. noguchii*, serogroup Panama, serovar panama	171	In MLST Home data base	KJ701741	
49	∗GIMC2035:Celledoni	*L. weilii*, serogroup Celledoni, serovar celledoni	185	In MLST Home data base	KJ701746	
50	∗GIMC2036:Sarmin	*L. weilii*, serogroup Sarmin, serovar sarmin	191	In MLST Home data base		
51	∗GIMC2032:CZ299U	*L. santarosai*, serogroup Pomona, serovar tropica	208	338		
52	GIMC2001:Bairam-Ali	*L. *sp., serogroup Bairam-Ali, serovar bairam-ali		KJ676852-KJ676858	KJ701604	KJ701750
53	∗GIMC2055:Lyme	*L. inadai*, serogroup Lyme, serovar lyme			KJ701743	
54	GIMC2056:EMJH 86	*L. inadai*, serovar lyme			KJ701744	
55	GIMC2057:Enr 88	*L. inadai*, serogroup Lyme-Detroit, serovar lyme-detroit			KJ701745	
56	∗GIMC2060:Sao Paulo	*L. yanagawae*, serogroup Semaranga, serovar sao paulo			KJ701747	
57	GIMC2058:LT-8	*L. biflexa*, serovar patoc			KJ701748	
58	GIMC2059:GR	*L. biflexa*, serovar andamana			KJ701749	
59	DSM 21527	*Turneriella parva *		NC_018020	CP002959	CP002959
60	80–412	*L. alstoni*, serovar pingchang			NZ_AOHD02000041.1	NZ_AOHD02000066.1
61	79601	*L. alstoni*, serovar sichuan				AY631881
62	L 60	*L. alexanderi*, serovar manhao 3			NZ_AHMT02000060.1	
63	A23	*L. alexanderi*, serovar manzhuang				AY996803.1
64	A85	*L. alexanderi*, serovar mengla				DQ991481.1
65	M 6901	*L. alexanderi*, serovar nanding				AY996804
66	Mus 127	*L. borgpetersenii*, serovar ballum			EU747302	
67	Lely 607	*L. borgpetersenii*, serogroup Sejroe, serovar hardjo				FJ154586
68	L550	*L. borgpetersenii*, serovar hardjo-bovis			CP000348.1	
69	JB197	*L. borgpetersenii*, serovar hardjo-bovis			CP000350	
70	Lely 607	*L. borgpetersenii*, serovar hardjo-bovis			EU747305	
71	Veldrat Batavia 46	*L. borgpetersenii*, serovar javanica				AY887899.1
72	M84	*L. borgpetersenii*, serovar sejroe			EU747311	
73	Perepelitsin	*L. borgpetersenii*, serovar tarassovi			EU747307	JQ988861.1
74	Whitticombi	*L. borgpetersenii*, serovar whitticombi			EU747314	
75	Mini-CTG	*L. borgpetersenii*, serovar no data				JQ765635.1
76	Ballico	*L. interrogans*, serovar australis				FJ154556.1
77	Akiyami A	*L. interrogans*, serovar autumnalis				AM050580.1
78	Jez-bratislava	*L. interrogans*, serovar bratislava			EU747300	
79	Mallika	*L. interrogans*, serovar bulgarica				AY996792.1
80	Hond Utrecht IV	*L. interrogans*, serovar canicola				FJ154561.1
81	Fiocruz L1-130	*L. interrogans*, serovar copenhageni	17	AE016823.1	AE016823.1	
82	Djasiman	*L. interrogans*, serovar djasiman			EU747312	FJ154550.1
83	Hardjo_DB33	*L. interrogans*, serovar hardjo				JQ988854.1
84	RGA	*L. interrogans*, serovar icterohaemorrhagiae				NR_029361.1
85	Kremastos	*L. interrogans*, serovar kremastos				AY461868.1
86	IPAV	*L. interrogans*, serovar lai			CP001221	
87	LT 398	*L. interrogans*, serovar manilae				FJ154545.1
88	Hond HC	*L. interrogans*, serovar medanesis				DQ991471.1
89	Pomona	*L. interrogans*, serovar pomona			EU747306	AY996800.1
90	Salinem	*L. interrogans*, serovar pyrogenes				FJ154552.1
91	Vleermuis	*L. interrogans*, serovar schueffneri			EU747313	
92	Szwajizak	*L. interrogans*, serovar szwajizak				DQ991466.1
93	3705	*L. interrogans*, serovar wolffi			EU747308	
94	Zanoni	*L. interrogans*, serovar zanoni				DQ991473
95	Bataviae_DB59	*L. interrogans*, serovar no data				JQ988841.1
96	Agogo	*L. kirschneri*, serovar agogo				DQ991476.1
97	Bafani	*L. kirschneri*, serovar bafani				DQ991477.1
98	1051	*L. kirschneri*, serovar bim				AY996802.1
99	Butembo	*L. kirschneri*, serovar butembo				Q991478.1
100	3522 C	*L. kirschneri*, serovar cynopteri				FJ154546.1
101	Moskva V	*L. kirschneri*, serovar grippotyphosa			EU747301	
102	Kambale	*L. kirschneri*, serovar kambale				AY461878.1
103	5621	*L. kirschneri*, serovar pomona				FJ154559.1
104	Wumalasena	*L. kirschneri*, serovar ratnapura				DQ991479.1
105	Bejo-Iso9	*L. kmetyi*, serovar malaysia		AHMP02000003	AHMP02000003	NR_041544.1
106	Hook	*L. noguchii*, serogroup Australis			EU349504	
107	Bonito	*L. noguchii*, serogroup Autumnalis			EU349501	
108	Caco	*L. noguchii*, serogroup Autumnalis			EU349498	
109	Cascata	*L. noguchii*, serogroup Bataviae			EU349502	
110	LSU 1945	*L. noguchii*, serogroup Louisiana			EU349505	
111	1348U	*L. noguchii*, serovar claytoni				DQ991498.1
112	1996K	*L. noguchii*, serovar cristobali				DQ991497.1
113	M7	*L. noguchii*, serovar huallaga				DQ991499.1
114	1011	*L. noguchii*, serovar nicaragua			EU349499	
115	LSU 2580	*L. noguchii*, serovar orleans			EU349500	
116	CZ 214 K	*L. noguchii*, serovar panama			EU349497	NR_043050.1
117	DB57	*L. noguchii*, serovar panama			EU349501	JQ988837.1
118	HS 616	*L. santarosai*, serovar alexi				FJ154585.1
119	Alice	*L. santarosai*, serovar alice				DQ991493.1
120	MAVJ 401	*L. santarosai*, serovar arenal			AHMU02000055	
121	LT 79	*L. santarosai*, serovar bakeri				FJ154589.1
122	LT 117	*L. santarosai*, serovar georgia				AY996805.1
123	CZ320	*L. santarosai*, serovar kobbe				DQ991495.1
124	1342	*L. santarosai*, serovar shermani				FJ154576.1
125	CZ390	*L. santarosai*, serovar weaveri				DQ991496.1
126	CBC613	*L. santarosai*, serovar no data			ANIH01000040	
127	ST188	*L. santarosai*, serovar no data			AOHA02000081	
128	MOR084	*L. santarosai*, serovar no data			AHON02000051	
129	2000030832	*L. santarosai*, serovar no data			AFJN02000030	
130	Celledoni	*L. weilii*, serovar celledoni				DQ991486.1
131	H27	*L. weilii*, serovar hekou				DQ991487.1
132	M39090	*L. weilii*, serovar langati				DQ991488.1
133	WB46	*L. weilii*, serovar sarmin				U12673.1
134	LT 89-68	*L. weilii*, serovar vughia				FJ154590.1
135	5399	*L. broomii*, serovar hurstbridge		AHMO02000008	AHMO02000008	AHMO02000008.1
136	BUT 6	*L. fainei*, serovar hurstbridge		AKWZ02000010	AKWZ02000010	NR_043049.1
137	BKID 6	*L. fainei*, serovar hurstbridge				AY996789.1
138	10	*L. inadai*, serovar lyme		AHMM02000025	AHMM02000015	AHMM02000015.1
139	VAR 010	*L. licerasiae*, serovar varillal		AHOO02000005	AHOO02000005	NR_044310.1
140	Khorat-H2	*L. wolffii*, serovar khorat		AKWX02000020		NZ_AKWX02000004.1
141	Patoc Ames	*L. biflexa*, serovar patoc		NC_010842	NC_010842	NC_010842
142	Patoc Paris	*L. biflexa*, serovar patoc		NC_010602	NC_010602	
143	CH 11	*L. biflexa*, serovar andamana				FJ154577.1
144	Veldrat Semarang 173	*L. meyeri*, serovar semaranga				AF157089.1
145	Went 5	*L. meyeri*, serovar hardjo		AKXE01000002	AKXE01000001	NZ_AKXE01000007.1
146	LT 11-33 ATCC 700639	*L. terpstrae*, serovar hualin		AOGW02000006	AOGW02000010	NZ_AOGW02000008.1
147	Waz Holland ATCC 700522	*L. vanthielii*, serovar holland		AOGY02000070	AOGY02000051	NZ_AOGY02000072.1
148	CDC; ATCC 43284	*L. wolbachii*, serovar codice		AOGZ02000014	AOGZ02000008	NR_043046.1
149	Sao Paulo ATCC 700523	*L. yanagawae*, serovar saopaulo		AOGX02000015	AOGX02000024	Z_AOGX02000022.1
150	Eri-1(T) DSM 26084(T) = JCM 18486(T)	*L. idonii*, serogroup Hebdomadis				AB721966

*The reference strains from GIMC.

**Table 2:** Physiological characteristics of the *Leptospira  *spp. strain Bairam-Ali.

Strain	Growth at temp (°C) of			Growth in the presence of	Lipase activity	Hemolytic activity against sheep erythrocytes
	11	30	37	8-Azaguanine 225 pg/mL		
Bairam-Ali	+	+	+	+	+	−

**Table 3:** Comparison *L.* spp. Bairam-Ali strain genome characteristics with most closely related *Leptospira* species.

*Leptospira* strain or species	Genome size, Mbp	GC%
*L*-BA∗	4,4	34,7
*L. interrogans *	4,56 ± 0,32	35,22 ± 0,27
*L. noguchii *	4,76 ± 0,16	35,63 ± 0,27
*L. terpstrae *	4,09	38,2
*L. meyeri *	4,15	38,05
*L. vanthielii *	4,23	38,9
*L. biflexa *	3,95	38,9

*L-BA: *Leptospira  *spp. Bairam-Ali.

## Abbreviations

L int: * L. interrogans*
L kir: * L. kirschneri*
L nog: * L. noguchii*
L wei: * L. weilii*
L ale: * L. alexanderi*
L bor: * L. borgpetersenii*
L san: * L. santarosai*
L als: * L. alstoni *
L kme: * L. kmetyi*
L lic: * L. licerasiae*
L wlf: * L. wolffii*
fai: * L. fainei*
bro: * L. broomii*
L ind: * L. inadai*
bif: * L. biflexa*
yan: * L. yanagawae*
mey: * L. meyeri*
ter: * L. terpstrae*
van: * L. vanthielii*
wlb: * L. wolbachii*
LBA: * L. *species Bairam-Ali
TURPA: * Turneriella parva*
INT: Intermediate species
NON-PATH: Nonpathogenic species.
