# Supplementary material for: The Characteristics of Ubiquitous and Unique Leptospira Strains from the Collection of Russian Centre for Leptospirosis
Source: Biomed Res Int. 2014 Sep 2;2014:649034. doi: 10.1155/2014/649034 (PMC4167648; doi:10.1155/2014/649034)
Supplement: Supplementary file 1 — Supplementary Table S1: The range of nucleotide sequences similarity (rrs data) between and within groups of Leptospiraceae samples. Supplementary Table S2: The range of nucleotide sequences similarity (rpoB data) between and within groups of Leptospiraceae samples. Supplementary Table S3: The range of nucleotide sequences similarity (MLST data) between and within groups of Leptospiraceae samples. Supplementary Figure S4: Phylogenetic tree of Leptospira species based on 16S rDNA sequences. [file 649034.f1.pdf]

S1. The range of nucleotide sequences similarity (rrs data) between and within groups of Leptospiraceae samples (L int - *L. interrogans*, L kir - *L. kirschneri*, L nog - *L. noguchii*, L wei - *L. weilii*, L ale - *L. alexanderi*, L bor - *L. borgpetersenii*, L san - *L.santarosai*, L als - *L. alstoni*, L kme - *L. kmetyi*, LBA - *L. species* Bairam-Ali, TURPA - *Turneriella parva*. INT - intermediate species, NON-PATH - non-pathogenic, PATH -pathogenic species) calculated by ClastalW2 (in %)

rrs max-min %	L ale	L int	L kir	L bor	L nog	L san	L wei	L kme	L als	PATH	INT	NON-PATH	LBA	TURPA
L ale	99.9-100													
L int	99.0-99.1	99.9-100												
L kir	99.0-99.2	99.7-99.9	99.9-100											
L bor	99.3-99.5	99.0-99.3	99.1-99.3	99.8-100										
L nog	98.8-98.9	99.2-99.3	99.3-99.3	99.0-99.1	99.9-100									
L san	98.7-99.0	98.7-98.9	98.7-99.0	99.0-99.3	98.9-99.0	99.8-100								
L wei	99.2-99.3	99.0-99.0	99.0-99.1	99.5-99.6	99.1-99.1	98.9-99.0	100							
L kme	98.7-98.8	99.0-99.0	99.0-99.0	98.9-99.0	99.0-99.0	98.5-98.7	98.8-98.8	100						
L als	97.8-98.9	98.1-99.1	98.2-99.1	98.0-98.9	98.1-99.0	97.6-98.6	97.9-98.7	98.6-99.4	99.1-99.1					
PATH	97.8-99.5	98.1-99.9	98.2-99.9	98.0-99.6	98.1-99.3	97.6-99.3	97.9-99.6	98.5-99.4	97.6-99.4	81.8-96.0				
INT	95.0-95.7	95.1-95.8	95.2-95.9	95.2-95.8	95.1-95.8	95.0-95.7	94.9-95.6	95.3-96.0	94.4-95.6	94.4-96.0	97.2-99.7			
NON-PATH	89.4-90.4	90.0-90.5	90.0-90.6	89.9-90.5	89.9-90.4	89.5-90.1	89.7-90.4	89.6-90.2	89.1-90.3	89.1-90.6	88.4-89.9	98.6-99.8		
LBA	90.3-90.4	90.9-91.0	90.8-90.8	90.7-90.8	90.7-90.7	90.7-90.8	90.7	90.7	90.0-90.7	90.0-91.0	90.8-91.1	91.7-92.7	100	
TURPA	82.2-82.4	82.4-82.4	82.4-82.5	82.7-82.7	82.5-82.5	82.5-82.5	82.3	82.5	81.8-82.4	81.8-82.7	82.7-83.3	81.4-81.9	82.0-82.0	100

S2. The range of nucleotide sequences similarity (rpoB data) between and within groups of Leptospiraceae samples (L int - *L. interrogans*, L kir - *L. kirschneri*, L nog - *L. noguchii*, L wei - *L. weilii*, L ale - *L. alexanderi*, L bor - *L. borgpetersenii*, L san - *L.santarosai*, L als - *L. alstoni*, L kme - *L. kmetyi*, LBA - *L. species* Bairam-Ali, TURPA - *Turneriella parva*. INT - intermediate species, NON-PATH - non-pathogenic, PATH -pathogenic species) calculated by ClastalW2 (in %)

rpoB max-min %	L ale	L int	L kir	L bor	L nog	L san	L wei	L kme	L als	PATH	INT	NON-PATH	LBA	TURPA
L ale	93.2-93.2													
L int	85.3-90.0	89.1-100												
L kir	89.5-92.2	89.5-92.2	99.3-100											
L bor	85.3-91.2	88.3-100	88.1-94.2	88.3-100										
L nog	88.3-92.2	86.7-90.4	91.2-93.0	87.1-91.6	94.0-100									
L san	86.3-90.2	86.3-91.8	89.8-97.7	86.7-93.6	90.2-93.4	88.7-99.5								
L wei	86.1-89.1	89.1-90	89.3-89.5	88.7-91.4	88.3-90.4	88.3-89.1	100							
L kme	87.3-90.0	89.3-91.4	92.4-92.4	89.1-92.2	91.2-93.4	90.8-92.2	89.8-89.8	100						
L als	86.5-89.8	87.5-89.3	89.1-89.3	87.3-90.2	88.7-90.0	88.9-89.3	87.7-87.7	89.8-89.8	100					
PATH	85.3-92.2	85.3-100	86.7-97.7	85.3-100	86.7-93.4	86.3-97.7	87.7-89.8	89.8-92.4	87.7-89.8	85.3-100				
INT	72.2-75.5	72.0-76.1	72.0-74.4	71.6-76.1	73.4-76.9	71.4-77.7	73.0-74.4	74.9-75.9	75.3-76.3	71.4-77.7	78.1-100			
NON-PATH	61.2-67.1	59.3-66.3	62.0-66.5	61.8-66.9	62.4-68.9	62.8-68.5	63.4-69.3	63.0-66.5	63.4-67.1	59.3-69.3	60.2-66.1	85.8-100		
LBA	67.1-67.1	65.7-70.0	55.6-56.0	65.7-69.5	65.7-67.7	66.7-67.3	65.7	67.5	67.1	65.7-67.5	64.0-68.5	62.4-65.9	100	
TURPA	53.3-57.9	53.5-58.9	67.5-67.5	53.5-59.1	56.6-59.5	55.4-60.3	56.4	58.3	57.9	56.0-58.3	55.6-58.3	56.4-58.5	57.2-57.2	100

S3. The range of nucleotide sequences similarity (MLST data) between and within groups of Leptospiraceae samples (L int - *L. interrogans*, L kir - *L. kirschneri*, L nog - *L. noguchii*, L wei - *L. weilii*, L ale - *L. alexanderi*, L bor - *L. borgpetersenii*, L san - *L.santarosai*, L als - *L. alstoni*, L kme - *L. kmetyi*, LBA - *L. species* Bairam-Ali, TURPA - *Turneriella parva*. INT - intermediate species, NON-PATH - non-pathogenic, PATH - pathogenic species) calculated by ClastalW2 (in %)

MLST max-min %	L ale	L int	L kir	L bor	L nog	L san	L wei	L kme	L als	PATH	INT	NON-PATH	LBA	TURPA
L ale	98.5-99.9													
L int	81.8-82.6	97.5-99.9												
L kir	81.9-82.7	92.4-93.9	98.1-99.9											
L bor	93.0-93.4	81.3-82.2	81.6-82.4	98.8-99.9										
L nog	80.8-82.0	91.2-91.9	90.8-91.9	80.7-81.8	96.8-99.5									
L san	87.5-88.1	81.4-82.1	81.4-82.1	86.8-87.3	80.9-81.7	99.2-99.8								
L wei	93.2-96.3	81.2-82.3	81.5-82.3	90.0-92.7	80.5-81.8	87.1-87.7	94.2-99.9							
L kme	83.6-84.3	80.2-81.8	81.1-82.0	82.1-82.7	80.7-81.9	84.3-84.3	83.2-85.2	100						
L als	84.9-85.7	82.1-83.0	82.7-83.8	83.8-84.3	81.9-83.3	84.8-85.3	84.9-86.4	86.2	100					
PATH	80.8-96.2	69.6-73.3	81.4-93.9	80.7-93.4	80.5-91.9	80.9-88.1	80.5-96.3	81.0-84.2	82.6-85.6	80.5-99.9				
INT	70.9-72.5	69.6-72.4	70.2-72.6	70.7-72.1	69.7-72.3	70.5-72.2	71.6-73.3	71.7-73.0	70.5-72.6	70.0-72.8	74.2-91.4			
NON-PATH	67.3-68.6	67.6-70	67.5-69.8	66.9-68.0	67.3-69.6	66.3-68.6	66.7-68.7	66.2-67.9	67.0-68.2	67.2-68.9	66.2-68.2	81.8-90.3		
LBA	68.3-68.8	68.3-69.7	67.6-69.1	67.7-68.3	68.4-69.6	66.2-67.1	66.5-68.7	66.9	66.5	66.2-69.7	65.8-67.3	67.6-69.1	100	
TURPA	56.7-57.1	52.9-55.3	53.8-57.1	57.0-57.5	54.1-56.6	56.3-58.9	56.8-58.6	58.3	58.1	53.5-58.2	55.9-58.3	55.4-56.8	55.3	100

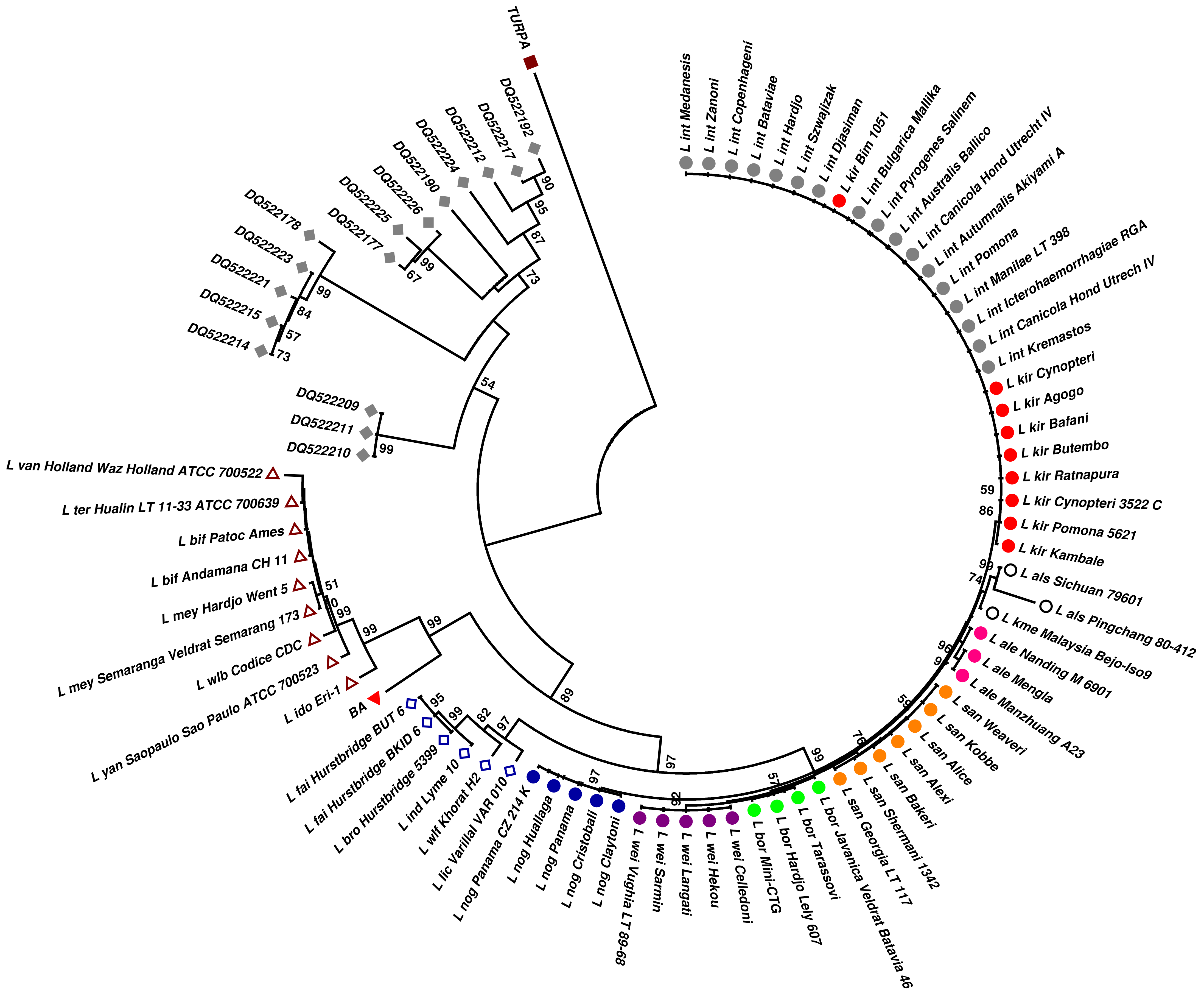

S4. Phylogenetic tree of Leptospira species based on 16S rDNA sequences.
Abbreviations: L int - L. interrogans, L kir - L. kirschneri, L nog - L. noguchii, L wei - L. weilii, L ale - L. alexanderi, L bor - L. borgpetersenii, L san - L.santarosai, L als - L. alstoni, L kme - L. kmetyi, L lic - L. licerasiae, L wlf - L. wolffii, fai - L. fainei, bro - L. broomii, L ind - L. inadai, bif - L. biflexa, yan - L. yanagawae, mey - L. meyeri, ter - L. terpstrae, van - L. vanthielii, wlb - L. wolbachii, BA - L. species Bairam-Ali, TURPA - Turneriella parva.
Different species or group of species have been marked in different colors. Gray diamonds indicate 16S rDNA sequences of leptospirae from the Peruvian Amazon, previously termed ''clade C'' [38]
